# Structure to function prediction of hypothetical protein KPN_00953 (Ycbk) from *Klebsiella pneumoniae* MGH 78578 highlights possible role in cell wall metabolism

**DOI:** 10.1186/1472-6807-14-7

**Published:** 2014-02-05

**Authors:** Boon Aun Teh, Sy Bing Choi, Nasihah Musa, Few Ling Ling, See Too Wei Cun, Abu Bakar Salleh, Nazalan Najimudin, Habibah A Wahab, Yahaya M Normi

**Affiliations:** 1 School of Biological Sciences, Universiti Sains Malaysia, 11800 USM Pulau Pinang, Malaysia; 2 School of Industrial Technology, Universiti Sains Malaysia, 11800 USM Pulau Pinang, Malaysia; 3 Enzyme and Microbial Technology Research Center (EMTECH), Faculty of Biotechnology and Biomolecular Sciences, Universiti Putra Malaysia, 43400 Serdang, Selangor, Malaysia; 4 School of Health Sciences, Health Campus, Universiti Sains Malaysia, 16150 Kubang Kerian, Kelantan, Malaysia; 5 Malaysian Institute of Pharmaceuticals and Nutraceuticals, Ministry of Science, Technology and Innovation, Blok 5-A, Halaman Bukit Gambier, 11700 Pulau Pinang, Malaysia

**Keywords:** KPN_00953, Hypothetical protein, Homology modeling, Peptidase M15_3 superfamily, Cell wall metabolism

## Abstract

**Background:**

*Klebsiella pneumoniae* plays a major role in causing nosocomial infection in immunocompromised patients. Medical inflictions by the pathogen can range from respiratory and urinary tract infections, septicemia and primarily, pneumonia. As more *K. pneumoniae* strains are becoming highly resistant to various antibiotics, treatment of this bacterium has been rendered more difficult. This situation, as a consequence, poses a threat to public health. Hence, identification of possible novel drug targets against this opportunistic pathogen need to be undertaken. In the complete genome sequence of *K. pneumoniae* MGH 78578, approximately one-fourth of the genome encodes for hypothetical proteins (HPs). Due to their low homology and relatedness to other known proteins, HPs may serve as potential, new drug targets.

**Results:**

Sequence analysis on the HPs of *K. pneumoniae* MGH 78578 revealed that a particular HP termed KPN_00953 (YcbK) contains a M15_3 peptidases superfamily conserved domain. Some members of this superfamily are metalloproteases which are involved in cell wall metabolism. BLASTP similarity search on KPN_00953 (YcbK) revealed that majority of the hits were hypothetical proteins although two of the hits suggested that it may be a lipoprotein or related to twin-arginine translocation (Tat) pathway important for transport of proteins to the cell membrane and periplasmic space. As lipoproteins and other components of the cell wall are important pathogenic factors, homology modeling of KPN_00953 was attempted to predict the structure and function of this protein. Three-dimensional model of the protein showed that its secondary structure topology and active site are similar with those found among metalloproteases where two His residues, namely His169 and His209 and an Asp residue, Asp176 in KPN_00953 were found to be Zn-chelating residues. Interestingly, induced expression of the cloned *KPN_00953* gene in lipoprotein-deficient *E. coli* JE5505 resulted in smoother cells with flattened edges. Some cells showed deposits of film-like material under scanning electron microscope.

**Conclusions:**

We postulate that KPN_00953 is a Zn metalloprotease and may play a role in bacterial cell wall metabolism. Structural biology studies to understand its structure, function and mechanism of action pose the possibility of utilizing this protein as a new drug target against *K. pneumoniae* in the future.

## Background

*Klebsiella pneumoniae* is a Gram-negative, rod-shaped bacterium that is widely distributed in soil and water [1] as well as the intestine, urethra and respiratory tract of mankind and other animals [2]. This opportunistic pathogen has been regarded as one of the major causes of respiratory and urinary tract infections, septicemia and the third-most-common bacterial cause of hospital-acquired pneumonia in immunocompromised patients [3]. Studies in Taiwan showed that this pathogen has the capacity to cause pyogenic liver abscess in human [4,5]. Similar cases have been observed in other countries as well, indicating that such medical infliction is not only confined to Taiwan per se and may potentially emerge as a global problem [6]. To add to this problem, *K. pneumoniae* strains which produce extended-spectrum beta-lactamases and are highly resistant to a spectrum of antibiotics are emerging worldwide [6]. These strains, also known as *K. pneumoniae* carbapenemases (KPC)-encoding strains, are often associated with nearly complete antibiotic resistance whereby failure and mortality rates related to pneumonia caused by this pathogen can reach up to 50% even with antibiotic therapy [7]. This makes treatments for this bacterium more difficult and has certainly created obstacles, no less danger, to public health.

Many components of the bacteria have been identified as pathogenic factors such as its capsular polysaccharide [8], yersiniabactin [9,10] and enterobactin [11]. All these pathogenic factors are well characterized in which their mechanisms of action and effects are established. Little is known, however, on the roles of poorly characterized biomolecules such as hypothetical proteins (HPs) of this pathogen. As their sequences and structures remain largely non-similar with other known proteins, HPs are often regarded as proteins of unknown functions [12] or orphan proteins [13]. It is important to note that a substantial fraction (up to 30 – 40%) of any sequenced bacterial genomes consist of genes which encode HPs [13]. This is certainly the case even in model organisms such as *Escherichia coli*, *Bacillus subtilis* or *Saccharomyces cerevisiae*[14]. Efforts to gain basic understanding on the roles and possible functions of HPs are crucial to close the gap between the “knowns” and the “unknowns”. This is especially important to fit and complete the genetic information puzzle of any living organisms, as well as to gain a ‘complete’ understanding of these organisms as biological systems as a whole [14].

As 25% of the complete genome sequence of *K. pneumoniae* MGH 78578 codes for HPs [15], it serves as a good mining pool for these proteins to be studied structurally and functionally. This effort is important particularly in substantiating the biological role and importance of HPs in the system of a pathogen. Improved understanding of these proteins may make them potential targets of antimicrobial drugs [14]. This present study highlights the *in silico* studies to characterize a HP, KPN_00953 (YcbK) from *K. pneumoniae* MGH 78578. The results revealed that KPN_00953 is a Zn-metalloprotease possibly related to the functions of the cell wall whereby its induced expression has interestingly changed the surface morphology of a lipoprotein-deficient *E. coli* JE5505 strain.

## Results

### Sequence analysis

The genome of *K. pneumoniae* MGH 78578 was obtained from NCBI website (Refseq: NC_009648) and thoroughly studied to identify the annotated proteins and HPs. A total of 1004 HPs were found in the genome of *K. pneumoniae* MGH 78578. Via pBLAST analysis [16] of the HPs against the non-redundant (NR) database, a particular hypothetical protein annotated as KPN_00953 (YcbK) gave more than 100 hits with values above the E-value threshold of 0.001. Majority of the top hits for this HP were also HPs and proteins with unknown functions (Table 1). Among these hits, a hypothetical lipoprotein from *Vibrio furnissii* NCTC 11218 and twin-arginine translocation pathway signal peptide showed high similarity to KPN_00953, up to 81% (data not shown) and 99%, respectively (Table 1). A search on the structures of these top hits in the Protein Data Bank (PDB) however, did not yield any result. In other words, no potential structural template among these top hits was found.

**Table 1:** Top 20 hits in BLAST search against NR database for KPN_00953

**Accession number**	**Title**	**Organism**	**SI (%)**	**SS (%)**	**E-value**
ZP_06550038.1 GI:290510668	Hypothetical protein HMPREF0485_02438	*Klebsiella sp. 1_1_55*	99	99	2e-113
YP_002918716.1 GI:238893982	Hypothetical protein KP1_1926	*Klebsiella pneumoniae NTUH-K2044*	99	100	4e-104
ZP_06014196.1 GI:262040974	Tat pathway signal sequence domain protein	*Klebsiella pneumoniae subsp. rhinoscleromatis ATCC 13884*	100	100	7e-103
YP_002239426.1 GI:206575875	Tat (twin-arginine translocation) pathway signal sequence domain/peptidase M15 family protein	*Klebsiella pneumoniae 342*	99	100	1e-102
ADO49107.1 GI:308749355	Protein of unknown function DUF882	*Enterobacter cloacae SCF1*	89	94	1e-100
NP_752993.1 GI:26246953	Hypothetical protein c1068	*Escherichia coli CFT073*	91	96	2e-95
ZP_05967118.1 GI:261339260	Hypothetical protein ENTCAN_05496	*Enterobacter cancerogenus ATCC 35316*	91	97	5e-95
YP_003613217.1 GI:296103071	Hypothetical protein ECL_02727	*Enterobacter cloacae subsp. cloacae ATCC 13047*	91	97	5e-95
CBK85464.1 GI:295096374	Uncharacterized protein conserved in bacteria	*Enterobacter cloacae subsp. cloacae NCTC 9394*	91	96	1e-94
YP_002382233.1 GI:218548442	Hypothetical protein EFER_1070	*Escherichia fergusonii ATCC 35469]*	90	95	4e-94
NP_309036.1 GI:15830263	Hypothetical protein ECs1009	*Escherichia coli O157:H7 str. Sakai*	92	96	6e-94
ZP_03066641.1 GI:194434378	Tat (twin-arginine translocation) pathway signal sequence domain/peptidase M15 family protein	*Shigella dysenteriae 1012*	92	96	6e-94
YP_001438498.1 GI:156934582	Hypothetical protein ESA_02416	*Cronobacter sakazakii ATCC BAA-894*	87	95	8e-94
NP_459971.1 GI:16764356	Hypothetical protein Ent638_1445	*Enterobacter sp. 638]*	90	96	1e-93
ZP_07097188.1 GI:300816969	Putative outer membrane protein	*Salmonella enterica subsp. enterica serovar Typhimurium str. LT2*	91	96	5e-93
NP_455482.1 GI:16759865	Tat pathway signal sequence protein	*Escherichia coli MS 107-1*	91	96	6e-93
ZP_02346752.1 GI:167553002	Hypothetical protein STY0998	*Salmonella enterica subsp. enterica serovar Typhi str. CT18*	90	96	1e-92
YP_001588730.1 GI:161614765	Putative exported protein, Tat-dependent	*Salmonella enterica subsp. enterica serovar Saintpaul str. SARA29*	90	95	1e-92
ZP_04561370.1 GI:237730889	Hypothetical protein SPAB_02517	*Salmonella enterica subsp. enterica serovar Paratyphi B str. SPB7*	90	95	1e-92
ZP_04561370.1 GI:237730889	Conserved hypothetical protein	*Citrobacter sp. 30_2*	90	96	2e-92

Conserved domains search on KPN_00953 using Uniprot [17] revealed that it contains a conserved domain found in the superfamily of M15_3 peptidases. Using this information, a similarity search was performed on KPN_00953 against all peptidases in the MEROPS Peptidase Database [18]. BLAST MEROPS results indicated that KPN_00953 shares similarity with many sub family M15A unassigned peptidases. This family of peptidase consists of metallopeptidases mostly specialized carboxypeptidases and dipeptidases such as Zn D-Alanyl-D-Alanine (D-Ala-D-Ala) carboxypeptidases. The biological functions of D-Ala-D-Ala carboxypeptidases are related to bacterial cell wall biosynthesis and metabolism [19-21].

To predict the possible function of KPN_00953, recently reported protein prediction methods comprising of FFPred [22], GOStruct [23], Argot2 [24], CombFunc [25] and PANNZER [26] were used. Out of this five prediction methods, GOStruct and PANNZER services were not available at the moment when the analyses were performed. Results obtained from FFPred indicated that KPN_00953 might be responsible for oxidation-reduction process with 0.952 probability. Since FFPred system is dedicated to assign gene ontology terms for eukaryotic protein sequences [22], the results obtained from the analysis of KPN_00953 using FFPred might not be accurate for a prokaryotic system. Analysis using CombFunc and Argot2 failed to predict any significant function for KPN_00953 although both highlighted that the protein consisted the Peptidase M_15 domain as well as a leucine rich domain. KEGG Orthology (KO) group analysis on KPN_00953 only annotated it as a hypothetical protein, unrelated to any KO group. Therefore, KPN_00953 could not be associated with any pathways based on the KO analysis.

**Figure 1:**
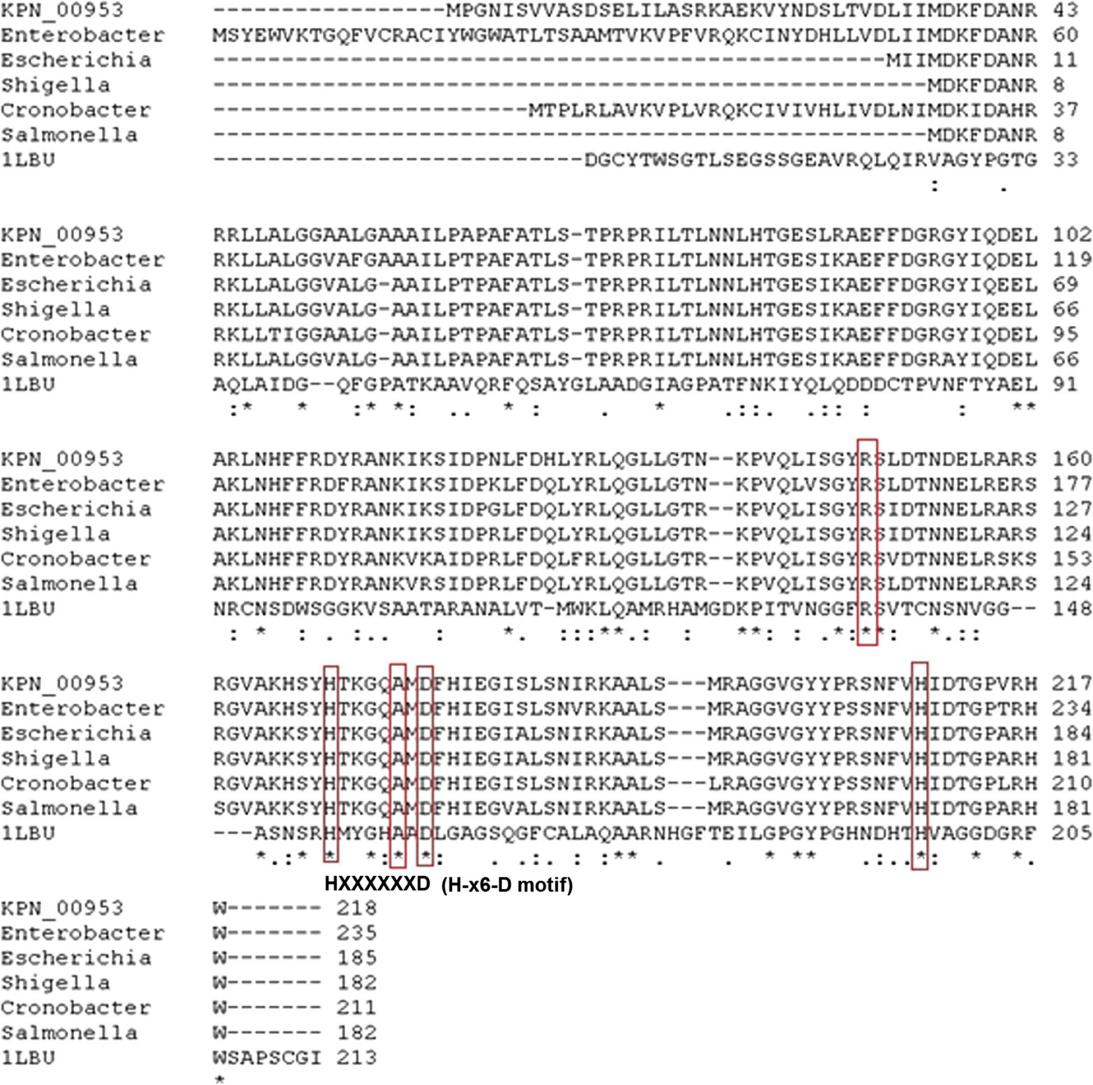
**MSA of KPN_00953 with 6 other conserved hypothetical proteins.** The two conserved His residues as well as Arg, Ala and Asp (highlighted in red) are believed to be responsible for Zn binding. The presence of the H-x6-D motif in the sequences is observed.

A multiple sequence alignment (MSA) analysis of KPN_00953 with sequences from six other organisms containing similar domain from the BLAST result was performed using ClustalW [27]. 33 residues from KPN_00953 were identical with these proteins (indicated with *, Figure 1). There were also 34 residues (indicated as :) which are conserved suggesting that the same chemical properties are shared albeit differences in sequence identity. Two Histidine residues i.e. His169 and His209, and an Aspartate residue i.e. Asp176 which are postulated to be involved in Zn chelation in well-characterized D-Ala-D-Ala carboxypeptidases, are interestingly found to be conserved here.

### Template selection

A search in the Protein Data Bank (PDB) on the potential structural template to be used to build the model of KPN_00953 was performed. No structure with high homology in PDB was detected for KPN_00953. However, since the MSA results confirmed the integrity of the putative Peptidase_M15_3 superfamily conserved domain in KPN_00953, this domain was used to search for such potential template in PDB instead. This resulted in the identification of one potential template, termed 1LBU with sequence identity of only 23%. 1LBU is a crystal structure of muramoyl-pentapeptide carboxypeptidase, a Zn^2+^ D-Ala-D-Ala carboxypeptidase from *Streptomyces albus*[20] which contain the particular Peptidase_M15_3 superfamily domain. 1LBU was also ranked top by Phyre2 search within CombFunc server as having the highest structural similarity KPN_0053. The results indicated that KPN_00953 might adopt similar fold and domain with 1LBU; namely the Hedgehog/D-Ala-D-Ala peptidase fold and Zn^2+^ D-Ala-D-Ala carboxypeptidase C-terminal catalytic domain. In fact, all the hits listed by Phyre2 contained the Hedgehog/D-Ala-D-Ala peptidase fold (Table 2). These results further stressed 1LBU as the best structural template for KPN_00953.

**Table 2:** Results extracted from Phyre2 analysis from CombFunc server

**Template**	**Confidence level (5)**	**Domain**
1LBU	100.0	Hedgehog/D-Ala-D-Ala peptidase
2 V09	98.1	Hydrolase/D-Ala-D-Ala peptidase
4MUR	97.9	Hydrolase/D-Ala-D-Ala peptidase
4JID	97.2	Hydrolase/D-Ala-D-Ala peptidase
2R44	95.8	Hedgehog/D-Ala-D-Ala peptidase

**Figure 2:**
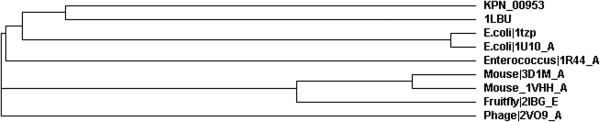
**Phylogenetic analysis of KPN_00953 with other protein structures from 8 other organisms including 1LBU (selected template).** The proteins were selected using SCOP hierarchy search. All the proteins contained the conserved Peptidase_M15_3 superfamily domain.

Phylogenetics analysis via SCOP search [28] was performed between KPN_00953 with other members of Peptidase_M15_3 superfamily to determine their degree of evolutionary relatedness. The analysis revealed that 1LBU was at a further clad from KPN_00953 (*Klebsiella sp*) in the cladrogram as compared to other organisms (Figure 2). Although the sequence identity of 1LBU compared with KPN_00953 is only 23%, it is evolutionary closer to *Klebsiella sp* based on its phylogenetic relationship. Moreover, the length of 1LBU is similar to KPN_00953 (Figure 1). Thus, 1LBU was selected as the template for homology modeling.

### Homology modelling of KPN_00953 and model validation

**Figure 3:**
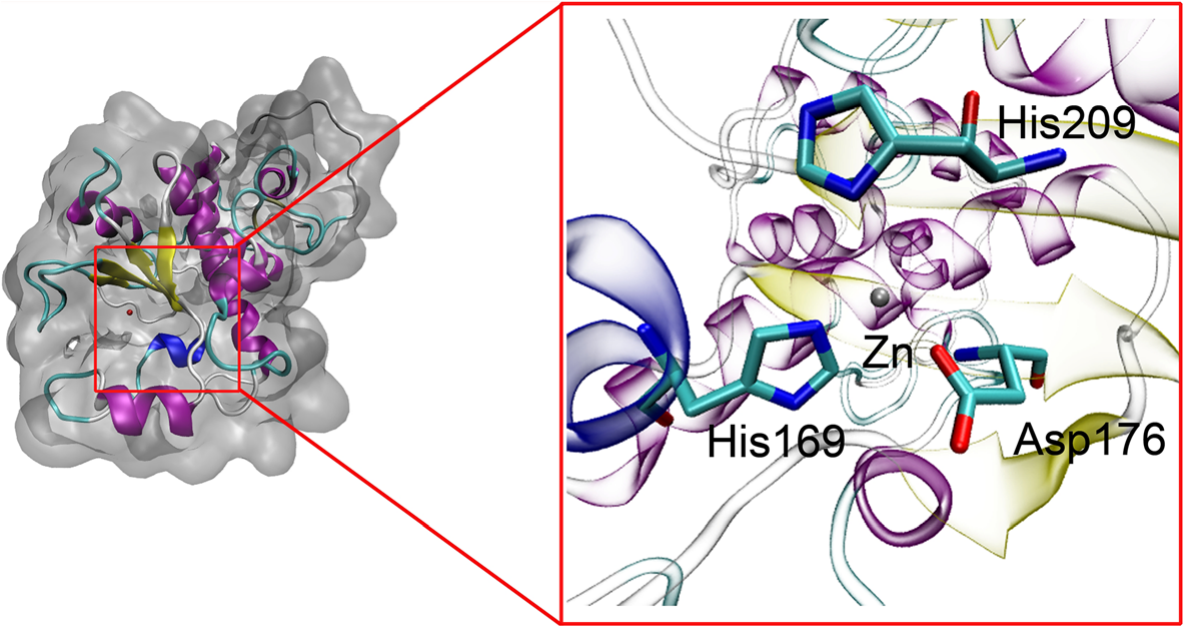
**The best model for KPN_00953 built using Modeller.** Conserved Zn-chelating residues such as His169, His209 and Asp176 are located within 4 Å from the Zn atom.

Homology modeling of KPN_00953 using MODELLER 9v8 [29] with 1LBU as the template randomly generated 20 models. The best model (with the lowest DOPE score) was subsequently validated using PROCHECK [30]. The Ramachandran analysis revealed that 96.6% of the amino acid residues reside in the most favourable and additional allowed regions (Table 3). The built model was further verified using Verify3D [31] and ERRAT [32]. Verify3D indicated that the built protein model scored 79.91%, suggesting compatibility between the amino acid sequence and the environment of the amino acid side chains in the model. ERRAT analysis on the protein model gave forth score of 63.285, a relatively acceptable assessment value on the arrangement of atoms with respect to one another in the protein model. In addition to these analyses, the compactness of the built model was also validated using ProQ protein quality prediction tool [33]. The result showed LG score of 1.304 and MaxSub score of 0.130, indicating that the built model of KPN_00953 is within the range of an acceptable model. Calculations of the interaction energy and Z-score using ProSA-Web [34] energy plot for each residue of the model gave forth value of −3.5 kcalmol^-1^. Based on these various structural evaluation results, the particular model can be accepted as a potential model for KPN_00953 (Figure 3).

**Table 3:** Statistical result of Ramachandran plot analysis for the best model in homology modeling

**Ramachandran plot (%)**	**Built KPN_00953 model ( *K. pneumoniae *) (%)**
Most favoured regions	84.2
Additional allowed regions	12.4
Generously allowed	2.6
Disallowed regions	1.1

### Structural and motif analyses

**Figure 4:**
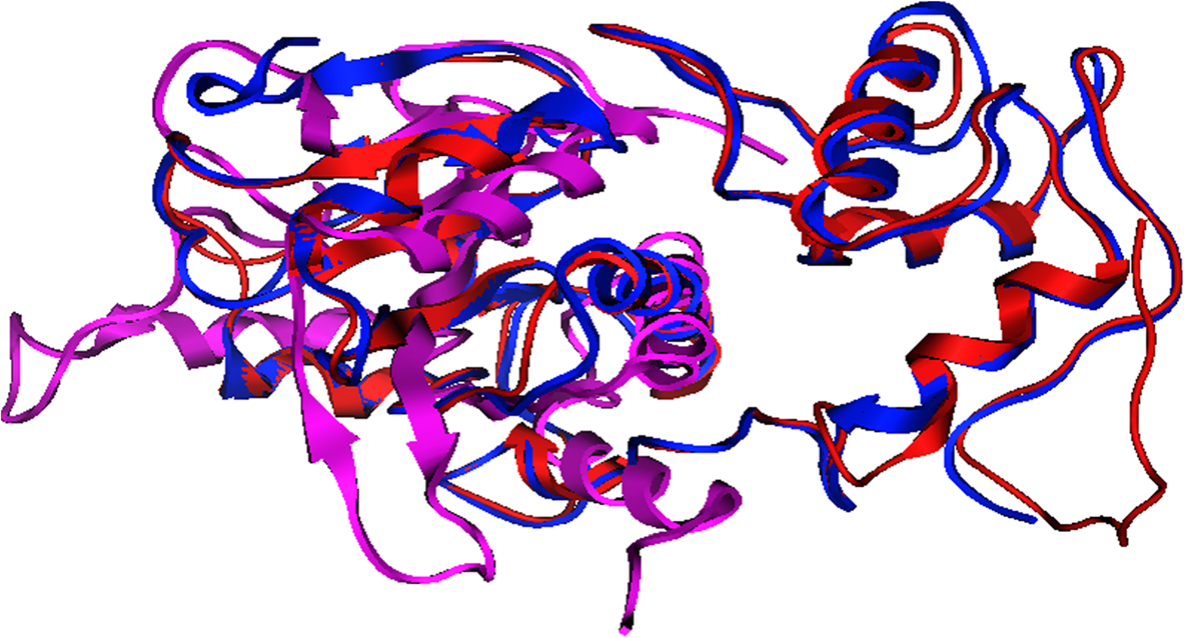
**Structural alignment of KPN_00953, 1LBU and 2VO9.** Four beta stranded regions are well aligned among KPN_00953 (red), 1LBU (blue) and 2VO9 (purple).

**Figure 5:**
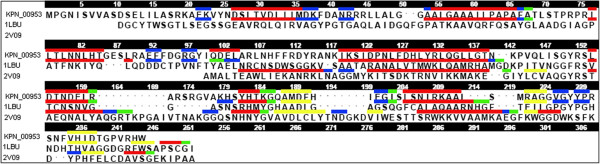
Secondary structure comparison of KPN_00953, 1LBU and 2VO9.

Structural alignment of KPN_00953 with the template 1LBU and 2VO9 (crystal structure of the distantly related L-alanoyl-D-glutamate endopeptidase domain of *Listeria* bacteriophage endolysin Ply500) [35] showed the integrity of the conserved domain. Structural analysis showed that the secondary structure, in particular the four beta stranded region and one single helix region, are well aligned (Figure 4). This is a unique secondary structure topology shared among metalloproteases [36,37]. The average RMSD between KPN_00953 and these two other structures is 5.42 Å. Further analysis on the secondary structure elements of the built model with 1LBU and 2VO9 using STRIDE [38] lends further support that these proteins share conserved secondary structure topologies (Figure 5).

Certain peptidases, particularly those of peptidoglycan hydrolases such as D-Ala-D-Ala metallopeptidases are believed to contain a Zn^2+^ ligand in most of the structures where the metal ion is coordinated by two histidines, an aspartate and a water molecule [36,37,39]. The presence of these active site residues is clearly observed in our built model, where His169, His209 and Asp176 are located exactly at Zn^2+^-chelating positions (Figure 3). Interestingly, these residues are also found to be highly conserved in certain hypothetical proteins from other organisms as well (Figure 1). It has been reported that other than these active site residues, there is another second conserved His residue two residues upstream of the His Zn^2+^ ligand [40]. This particular His residue, His166, was observed in the sequence of KPN_00953 where it is located two residues upstream of His169 (the Zn^2+^ ligand) (Figure 1).

The intactness of both the secondary structure topology and the three Zn^2+^ ligand-binding residues of the built model suggest that KPN_00953 may function as a cell wall (peptidoglycan)-hydrolyzing enzyme, similar to a few characterized Zn D-Ala-D-Ala metallopeptidases such as muramoyl-pentapeptide carboxypeptidase from *S. albus*[20] and VanX from *Enterococcus faecalis*[19]. Closer inspection on the sequence of KPN_00953 in comparison to the abovementioned proteins revealed that it does not contain the characteristic H-x-H motif which is predominantly found in nearly all D-Ala-D-Ala metallopeptidases, except VanX [40]. This motif is present in the sequence of 1LBU, the template used for the homology modeling of KPN_00953 (Figure 1). In the case of VanX, instead of the signature H-x-H motif it bears the E-x-x-H motif in its sequence [40]. This motif was absent also in the sequence of KPN_00953. However, KPN_00953 was found to possess the H-x (3–6)-D motif similar to MepA peptidase (Figure 1). Similar to the Zn D-Ala-D-Ala metallopeptidases stated above, MepA is a Zn-metalloprotein shown to be involved in cell wall related functions [41]. The only deviation to this similarity is the absence of the H-x-H motif in KPN_00953, which is reported to be present in MepA [40].

### Amplification and cloning of KPN_00953

**Figure 6:**
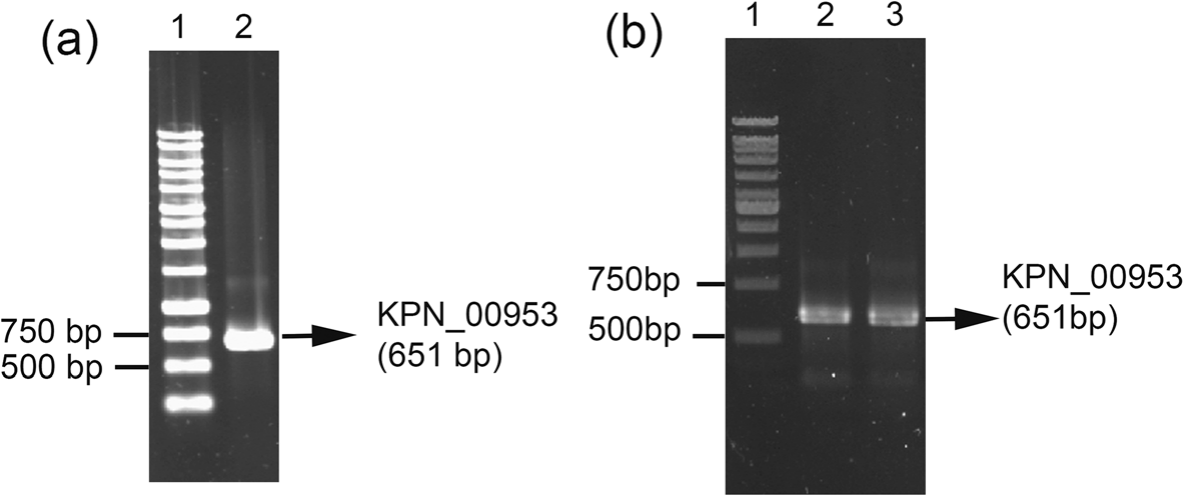
**Amplification of KPN_00953.** KPN_00953 amplicon (657 bp) amplified from **(a)** the genome of *K. pneumoniae* MGH 78578 (Lane 2) and **(b)***E. coli* JM109 transformants via colony PCR (Lanes 2 and 3).

To characterize further the possible function of KPN_00953, its Open Reading Frame (ORF) was amplified from the genome of *K. pneumoniae* MGH 78578 using specifically designed primers. A specific amplicon of 657 bp was obtained (Figure 6(a)). Cloning of this amplicon into pGEM®-7zf (+) was subsequently achieved, as confirmed from blue-white screening (data not shown), colony PCR (Figure 6(b)) and sequencing (data not shown).

### Altered cell surface morphology of *E. coli* JE5505 overexpressing KPN_00953

**Figure 7:**
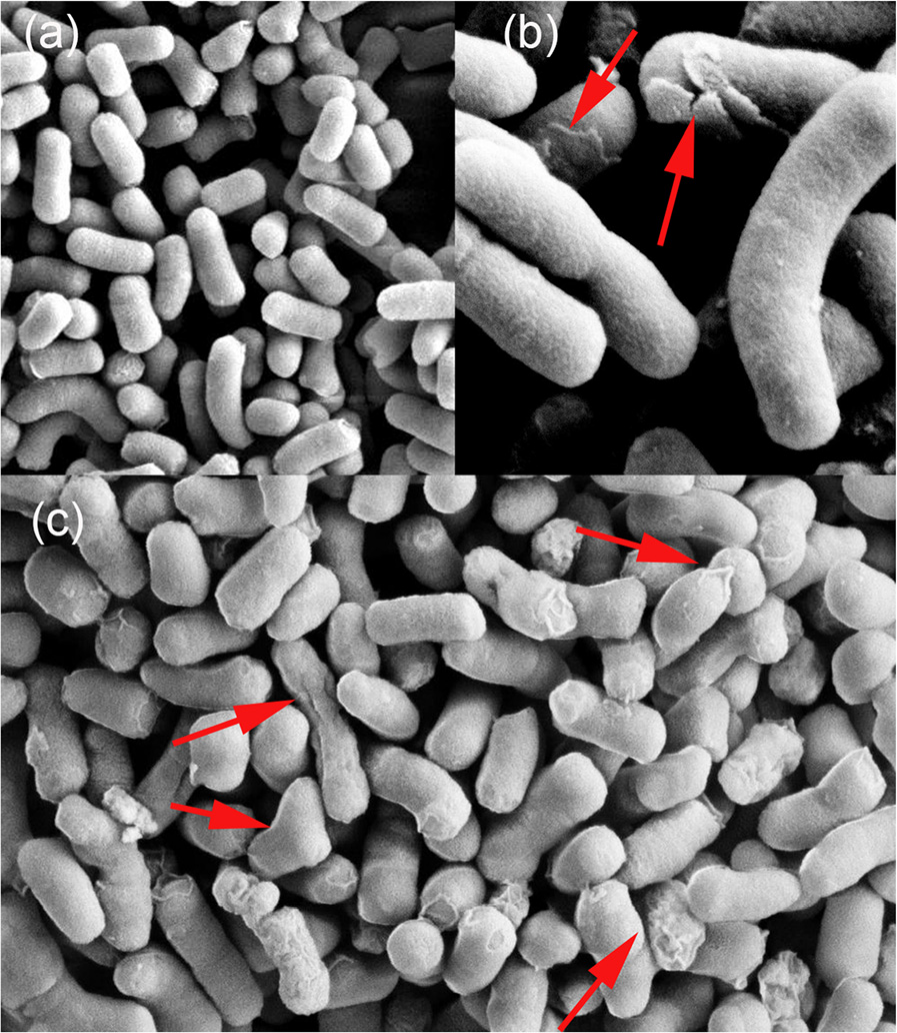
**Cell surface morphology of *E. coli *JE5505 cells.** Cell surface morphology of *E. coli* JE5505 cells **(a)** containing pGEM-7zf (+) plasmid (control) and those overexpressing KPN_00953 at **(b)** 200 nm and **(c)** 1 μm scale. Red arrows indicate material deposits on the surface of the cells.

Since homology modeling results point to the possibility of KPN_00953 having cell wall related metabolic functions i.e. peptidoglycan degradation, the effect of overexpressing this HP on cell surface morphology was investigated. For this purpose, the cloned *KPN_00953* construct was introduced into the lipoprotein-deficient *E. coli* JE5505 strain [42] and subsequently overexpressed via IPTG induction. Cells which overexpressed KPN_00953 appeared to have different surface morphology than cells which do not expressed this protein. They appear to be slightly smoother with flattened edges (Figure 7(c)), and some of them seem to have deposits on their surfaces (Figure 7(b) and (c)). In contrast, the cells which contained only the expression vector (control) have more well-defined and rougher surface texture (Figure 7(a)). Such alterations and deposits observed on the surface of the cells may suggest possible cell wall degradation by KPN_00953.

## Discussion

We have identified that HP KPN_00953 from *K. pneumoniae* MGH 78578 contains a well conserved domain belonging to the M15 superfamily of peptidases. Template identification based on this domain has led to the building of a 3D model of KPN_00953 via homology modeling using the crystal structure of muramoyl-pentapeptide carboxypeptidase (PDB id: 1LBU), a Zn D-Ala-D-Ala metallopeptidase from *S. albus*[20] as the template. The built model has been verified to be acceptable and topologically conserved with other available structures related to peptidases such as the L-alanoyl-D-glutamate endopeptidase domain of *Listeria* bacteriophage endolysin Ply500 (PDB id: 2VO9) [35]. Two His residues, His169 and His209, as well as an Asp residue, Asp176 of this model are postulated to be involved in Zn chelation (Figure 3) and are interestingly found to be conserved in other well characterized Zn-metalloproteases [36,37,39,40]. It is important to note that several members of Zn-metalloproteases act as peptidoglycan hydrolases; in which they are involved in cell wall metabolism.

The cell wall of bacteria contains peptidoglycan which is important in preserving and maintaining the structural integrity of the cell by withstanding turgor. It is closely linked to several physiological processes such as cell growth and division. Inhibition of its biosynthesis via the action of antiobiotics for instance as well as its degradation by lysozyme will result in cell lysis [21]. Peptidoglycan in general is made of alternating units of N-acetyl-glucosamine and N-acetyl-muramic acid that are linked via 1,4-glycosidic bonds. The muramyl residues serve as platforms for the attachment of short polypeptides which contain both L- and D-amino acids and typically have two D-Ala residues at the C terminus. These peptide components on the muramyl residues can be crosslinked by transpeptidation, which subsequently will result in a loss of the terminal D-Ala and strengthening of the bacterial cell wall [40,43].

Members of Zn-metalloproteases which have been characterized to be involved in peptidoglycan (cell wall) biosynthesis and metabolism include Zn D-Ala-D-Ala carboxypeptidases and dipeptidases [19]. Muramoyl-pentapeptide carboxypeptidase, which is used as a template in the homology modeling of KPN_00953, is a specific Zn D-Ala-D-Ala carboxypeptidase [20]. It was reported that this particular enzyme from *Streptomyces* hydrolyzes the C-terminal peptide bond of peptides of general structure R-D-Ala-D-Xaa. The lytic and extracellular characteristics of the enzyme brought about the suggestion that this enzyme is used by *Streptomyces* for fighting competitors in its ecological niche since it does not hydrolyze the *Streptomyces* peptidoglycan [20]. Another instance is the Zn-dependent D-Ala-D-Ala (amino) dipeptidase, VanX. This peptidase reduces the cellular pool of the D-Ala-D-Ala dipeptide so that only the D-Ala-D-lactate peptidoglycan chain precursors are produced and incorporated into the cell wall instead of the former. The modified peptidoglycan reportedly exhibited a 1,000-fold decrease in affinity for vancomycin. This feature is responsible in conferring antibiotic resistance to pathogenic bacteria such as vancomycin-resistant Enterococci (VRE) [19]. Both of these proteins were reported to be similar and structurally related [44,45] despite the differences in their sequences. The muramoyl-pentapeptide carboxypeptidase from *S. albus*, like nearly all Zn D-Ala-D-Ala metallopeptidases, contains the H-x-H motif. VanX, on the other hand, contains the E-x-x-H motif making it an exception among the D-Ala-D-Ala metallopeptidases [40].

In the case of KPN_00953, it does not possess both of these characteristic motifs. Instead, it contains the H-x (3–6)-D motif, similar to MepA peptidase [40,41]. However, it is important to stress that MepA contains as well the characteristic H-x-H motif [40,41] which KPN_00953 lacks. In terms of molecular function, MepA is reported to cleave D-alanyl-meso-2,6-diamino-pimelyl peptide bonds in *E. coli* peptidoglycan and is classified as a peptidase of unknown fold and catalytic class due to its low sequence similarity with other peptidases [41]. KPN_00953 resembles MepA in this respect in which KPN_00953 is shown to be related to the subfamily M15A of unassigned peptidases from BLAST MEROPS scan. In contrast to MepA however, KPN_00953 could be assigned to a subfamily or catalytic class of peptidases, namely the M15A subfamily of peptidase. As this subfamily of peptidases consists of a number of characterized members such as Zn D-Ala-D-Ala carboxypeptidases, functional inference of KPN_00953 could be made based on this information. Although KPN_00953 contains the H-x (3–6)-D motif of MepA, conserved domain analysis result seems to relate KPN_00953 more to Zn D-Ala-D-Ala metallopeptidase in general, excluding VanX. Hence, the presence of the H-x (3–6)-D motif of MepA in KPN_00953 may occur by chance.

It is important to note that whilst both domain analysis and homology modeling of KPN_00953 revealed the conservation of important domains, Zn-chelating residues and secondary structure topologies to D-Ala-D-Ala carboxypeptidase, sequence similarity search revealed that this particular HP is also related to the twin-arginine translocation (Tat) pathway signal sequence. Tat pathway is a protein transport system for the export of folded proteins [46]. Proteins which are targeted to the Tat pathway are exported to the cell envelope or to the extracellular space by tripartite N-terminal signal peptides and Tat translocase, which are found in the cytoplasmic membrane [47]. However, it is important to note that KPN_00953 lacks the consensus twin-arginine motif, (S/T)-R-R-x-F-L-K, which is reported to be present in all types of bacterial signal peptides [48,49], despite the sequence similarity mentioned above. In addition to this, KPN_00953 does not contain signal peptide sequence as revealed by analyses using various signal peptide detection software such as SignalP 4.0 [50], Signal–3 L [51], iPSORT [52] and SOSUISignal [53]. Hence, this omits the possibility that KPN_00953 is functionally related to Tat pathway.

Further inference on the possible function of KPN_00953 was attempted by cloning and expressing its gene in *E. coli* JE5505 strain which is deficient in lipoprotein production. Induced production of KPN_00953 in this particular strain changed the morphology of the cells. They appeared to be smoother with less defined edges with some having deposits on their surfaces. Cells which did not produce the cloned KPN_00953 protein appeared to be rougher with defined edges (Figure 7). This observation highlights the possibility of KPN_00953 to be involved in the functions of the cell wall, similar to other characterized peptidoglycan-hydrolyzing Zn metallopeptidases.

## Conclusions

Based on the three-dimensional model, domain and residues conservation of KPN_00953 to D-Ala-D-Ala carboxypeptidase, we hypothesize that KPN_00953 adopts the functionality as a metallopeptidase with an important role in cell wall metabolism. This is further supported by the altered surface morphology of *E. coli* JE5505 cells overexpressing KPN_00953. The mechanism as to how KPN_00953 brings about these changes is worthy to be investigated in the near future. This can be achieved via gene-knockout and structural biology studies to understand its structure, function and mechanism of action. Such efforts will undoubtedly address the possibility of utilizing this protein as a new drug target against *K. pneumoniae* in the future.

## Methods

### Bacterial strains and plasmids used

*K. pneumoniae* MGH 78578 was purchased from American Type Culture Collection (ATCC number: 700721). *Escherichia coli* JM109 [*end*A1, *rec*A1, *gyr*A96, *thi*, *hsd*R17 (r_k_–, m_k_+), *rel*A1, *sup*E44, Δ (lac-proAB)] was used for standard cloning purposes. For microbial plate assay of the expressed HP on cell surface morphology, a lipoprotein deletion mutant, *Escherichia coli* JE5505 [*Δ (gpt-proA)62*, *lacY1*, *tsx-29*, *glnV44*(AS), *galK2*(Oc), *λ*^*-*^, *Δlpp-254*, *pps-6*, *hisG4*(Oc), *xylA5*, *mtl-1*, *argE3*(Oc), *thi-1*] [33] was used (purchased from *E. coli* Genetic Resource Center, Yale University). pGEM-7Zf (+) (Promega) was used as an expression vector to express the cloned *KPN_00953* gene.

### Sequence analysis, model building and validation

The complete genome sequence of *K. pneumoniae* MGH 78578 was obtained from NCBI website [Refseq: NC_009648]. The sequences for HPs of the pathogen were selected and analyzed preliminarily using Uniprot [17]. KPN_00953 (YcbK) was selected based on the presence of the conserved M15 peptidase domain. KPN_00953 was subjected to a series of BLAST [16] search against non-redundant database (NR) and Protein Data Bank (PDB). FFPred [22], GOStruct [23], Argot2 [24], CombFunc [25] and PANNZER [26] were used to predict the possible function of KPN_00953. SignalP 4.0 [50], Signal–3 L [51], iPSORT [52] and SOSUISignal [53] were used to determine the possible presence of signal peptide in the sequence. Multiple sequence alignment (MSA) of KPN_00953 with six other proteins sequences from other organisms was later performed with ClustalW [27]. These proteins were selected from the list of potential hits in BLAST result which contained similar domain with KPN_00953. 1LBU [20] was selected as the template for homology modeling of KPN_00953 using MODELLER 9v8 [29]. Twenty models were generated randomly and model with the best Discrete Optimized Potential Energy (DOPE) score was selected and subsequently verified using PROCHECK [30], Verify3D [31], ERRAT [32], ProQ [33] and ProSA-Web [34] energy plot.

### Genomic DNA extraction

Genomic DNA extraction of *K. pneumoniae* MGH 78578 was performed using Wizard® Genomic DNA Purification Kit (Promega). The integrity and quality of the genomic DNA extracted were analyzed via agarose gel electrophoresis.

### Amplification of KPN_00953 Open Reading Frame (ORF)

*KPN_00953* (*ycbK*) gene was amplified by Polymerase Chain Reaction (PCR) using: 5’-TTAAGCTTTTGCCGGGCAACATCTCG-3’ (forward primer) and 5’-TCTTGGATCCTTACCAGTGCCTTACGGG-3 (reverse primer). Underlined sequences refer to incorporated *Hind*III and *Bam*HI restriction sites, respectively. The PCR reaction mixture (50 μl) contained Go Taq® Flexi Buffer (1X), dNTP mixture (0.2 mM), MgCl_2_ (1.0 mM), forward and reverse primers (1.0 μM each), genomic DNA template (0.5 ng) and 0.25U Go Taq® polymerase (Promega). A cycle of 95°C denaturation for 5 minutes, followed by 30 cycles of denaturation at 95°C for 1 minute, annealing at 55°C for 1 minute and extension at 72°C for 1 minute, and lastly further extension at 72°C for 5 minutes were performed. The amplicon was subsequently analyzed and purified using the QIAquick PCR Purification Kit (QIAGEN).

### Cloning of KPN_00953 amplicon into plasmid vector

The KPN_00953 amplicon and pGEM®-7zf (+) plasmid vector were subjected to *Bam*HI/*Hind*III double digestion at 37°C for 5 hours and subsequently analyzed by gel electrophoresis followed by purification. The products were later ligated using T4 DNA ligase (New England Biolabs) at 16°C, overnight. Ligated products were then transformed into *E. coli* JM109 via the TSS transformation method [54]. The presence of the recombinant plasmid constructs was verified via blue-white screening, colony PCR and sequencing. Upon positive verification of the desired construct, it was transformed into *E. coli* JE5505 (a lipoprotein-deficient strain).

### Cell surface observation of E. Coli JE5505 overexpressing KPN_00953 hypothetical protein by scanning electron microscope (SEM)

*E. coli* JE5505 cells harboring the cloned *KPN_00953* gene were cultivated in 5 ml LB broth supplemented with 100 μg/ml ampicillin and 0.1 mM IPTG for 16 hours at 180 rpm and 37°C. As the pGEM®-7zf (+) plasmid contains a T7 promoter, IPTG was used to induce the expression of *KPN_00953* in *E. coli* JE5505. Cells were harvested by centrifugation at 2000 rpm for 15 minutes at room temperature and were resuspended with 300 μl of McDowell-Trump fixative prepared in 0.1 M phosphate buffer (pH7.2) for at least 2 hours. The cells were centrifuged at the same speed and duration before being resuspended again in 500 μl 0.1 M phosphate buffer. This step was repeated once and followed by resuspension in 1% osmium tetroxide prepared in the phosphate buffer for 1 hour. The cells were centrifuged and finally resuspended in distilled water. These steps were repeated thrice to ensure the pellet was properly washed. Next, dehydration steps using increasing concentrations of ethanol were performed on the sample: 50% ethanol for 10 minutes, 75% ethanol for 10 minutes, 95% ethanol for 10 minutes and 100% ethanol for 10 minutes (twice). The final dehydration process was performed using hexamethyldisilazane. The centrifugation speed and duration employed for each of the dehydration step were the same as described above. After hexamethyldisilazane was decanted, the cells in the tube were left in the desiccators to be air-dried at room temperature. They were then mounted onto an SEM specimen stub with a double-sided sticky tape and coated with gold to be viewed under SEM.

## Competing interests

The authors declare that they have no competing interests.

## Authors’ contributions

TBA carried out the molecular cloning, expression and characterization studies. SBC carried the *in silico* studies related to sequence homology and evolutionary relatedness analyses as well as homology modeling. NM carried out the localization, signal peptide and sequence motifs analyses as well as sequencing analysis of the cloned plasmid. All three drafted the manuscript. FLL, STWC, NN, ABS, HAW and YMN revised and proofread the manuscript. YMN and HAW conceived the study and participated in its design and coordination. NN, ABS, FLL and STWC gave technical advice to the study. All authors read and approved the final manuscript.
